# Validation of the Revised Version of the Social Cognitive Theory-Based Scale for Factors Influencing Eating Behavior in Adolescents

**DOI:** 10.3390/healthcare14142229

**Published:** 2026-07-22

**Authors:** Ana Silvia Flores-Vázquez, Norma Patricia Rodriguez-Rocha, Karina Franco-Paredes, Gabriela Macedo-Ojeda

**Affiliations:** 1 Departamento de Alimentación y Nutrición, Centro Universitario de Ciencias de la Salud, Universidad de Guadalajara, Sierra Mojada 950, Independencia, Guadalajara 44340, Jalisco, Mexico; ana_flores_v@outlook.es (A.S.F.-V.); norma.rodriguez@academicos.udg.mx (N.P.R.-R.); 2 Instituto Regional de Investigación en Salud Pública, Centro Universitario de Ciencias de la Salud, Universidad de Guadalajara, Sierra Mojada 950, Independencia, Guadalajara 44340, Jalisco, Mexico; 3 Departamento de Promoción, Preservación y Desarrollo de la Salud, Centro Universitario del Sur, Universidad de Guadalajara, Avenida Enrique Arreola Silva 883, Centro, Ciudad Guzmán 49000, Jalisco, Mexico; karina.franco@cusur.udg.mx; 4 Instituto de Investigación en Ciencias Biomédicas, Centro Universitario de Ciencias de la Salud, Universidad de Guadalajara, Sierra Mojada 950, Independencia, Guadalajara 44340, Jalisco, Mexico

**Keywords:** scale, social cognitive theory, nutrition, adolescents, reliability, validity

## Abstract

**Background/Objectives**: Social Cognitive Theory (SCT) constructs are associated with adolescents’ eating habits. Validated surveys are needed to assess these constructs (in different populations and languages), considering both personal and socio-environmental factors, including peer influence. Therefore, the objective of this study was to validate the SCT-based Scale for Factors Influencing Eating Behavior in Adolescents (SCT-FIEBA), in Spanish. **Methods**: The scale was administered to 705 high school students from two schools in the Guadalajara Metropolitan Area, Mexico, with randomized and stratified selection. Two weeks later, 43 participants completed the retest. Construct validity was assessed using exploratory factor analysis (EFA) and confirmatory factor analysis (CFA); internal consistency was assessed using Cronbach’s alpha and McDonald’s omega; and temporal stability was assessed using the Intraclass Correlation Coefficient (ICC), with 95% confidence intervals (CIs). Additionally, factor patterns influencing eating behavior were identified using hierarchical cluster analysis and discriminant analysis. **Results**: EFA (*n* = 353) showed an adequate structure with seven or eight factors (with 34 items). However, CFA (*n* = 352) showed a better fit with eight factors and 33 items (RMSEA = 0.073, SRMR = 0.083, CFI = 0.91, TLI = 0.90) than the other models. The scale’s internal consistency was excellent (Cronbach’s alpha = 0.92, CI 0.92–0.93; McDonald’s omega = 0.93, CI 0.92–0.93), with all sections scoring from moderate to excellent. The reproducibility indices were good for the whole scale (ICC = 0.70, CI 0.50–0.82) and four sections; the other four presented moderate values. It is recommended to verify reproducibility in a larger sample. Hierarchical cluster analysis yielded better results with four patterns influencing eating behavior based on SCT, and the discriminant analysis results were satisfactory (Wilks’ lambda = 0.108; 87% of cases correctly classified). **Conclusions**: The SCT-FIEBA demonstrated validity and reliability for assessing SCT constructs applied to eating behavior among high school adolescents in a Mexican population, as well as for identifying four distinct psychosocial patterns that influence eating behavior.

## 1. Introduction

Adolescence is a critical period of growth and development [1]; consequently, health-related habits—particularly dietary patterns—established during this stage have long-term implications for health in adulthood. However, the current environment in which adolescents develop promotes inadequate nutrition, resulting in persistent nutritional deficiencies and a global increase in the prevalence of childhood overweight and obesity [2,3]. According to the World Health Organization (WHO), in 2022, more than 390 million children and adolescents were overweight, of which 160 million suffered from obesity [3].

Overweight and obesity, as well as their comorbidities, are preventable if health promotion actions are implemented from the early stages of life [3]. Health promotion aims to support people in adopting healthy habits. Therefore, it is essential to elucidate the determinants influencing the adoption of both healthy and risky behaviors during this period. In this sense, theories or models have emerged to explain the causes of health-related behaviors. These include the Health Belief Model, the Transtheoretical Model, the Theory of Planned Behavior, the Protection Motivation Theory, and Social Cognitive Theory (SCT), among others [4].

SCT, developed by Albert Bandura, is widely recognized for its efficacy in adolescent research [4], as it provides a comprehensive framework for explaining the acquisition of behaviors, attitudes, and beliefs from a socio-environmental perspective. Observational studies have shown that the constructs of this theory are associated with adolescents’ health behaviors, including physical activity [5], aggression [6], and eating habits [7]. Furthermore, several SCT-based intervention studies have confirmed their effectiveness in changing eating behavior [8]. Therefore, it was the behavioral theory selected for the development of this study.

SCT is based on reciprocal determinism: the bidirectional interaction between health behavior, personal factors (cognitions, beliefs, skills), and socio-environmental factors. That is, both personal and environmental factors influence an individual’s behavior, and this, in turn, can modify their environment and personal aspects. Similarly, environmental factors can affect an individual’s beliefs and cognitions, and vice versa [9].

Among personal constructs, self-efficacy is a key determinant, defined as the self-perceived capacity to learn or develop an action. This is closely linked to behavioral self-regulation, which involves monitoring and adjusting one’s behavior in accordance with personal goals or internal standards. In turn, outcome expectations are the expected results of actions, shaped by prior personal experiences and observations of role models. In addition, values (outcome valuation)—that is, the value placed on a behavior and its expected outcomes—are important because they influence motivation to act [9]. These two constructs, while closely related, refer to two distinct aspects: the first asks, “What results do I expect from modifying my behavior?” and the second asks, “How important are those results to me?”.

Some social–environmental constructs include facilitators (socio-environmental stimuli to carry out a behavior) and learning models (the observation of people, physical or virtual, who exhibit a specific behavior, attitude, or skill that can be imitated); in this construct, the effectiveness of peer models is particularly significant, as perceived similarity enhances both the self-efficacy and motivation required to adopt specific behaviors. In addition, social support or persuasion play important roles in increasing self-efficacy [9].

Given the relevance of this theory and its constructs to the adoption and practice of healthy habits, validated scales are needed to quantify and describe the extent to which these constructs manifest in individuals or populations in specific behaviors (e.g., diet, physical activity). These scales must be adapted and validated for a specific population group, and must include reliability measures (such as internal consistency and temporal stability) and validity measures [10]. The use of validated scales enables the planning and implementation of health interventions tailored to the target population and the assessment of their effectiveness in improving relevant theoretical constructs and in promoting the adoption of healthy behavior.

The Spanish-language Questionnaire on Adolescent Eating Behavior based on the SCT (SQAEB-SCT) was validated by Flores-Vázquez et al., in 2020 [11], among adolescents (aged 12–15 years). This first version of the scale is based on the English-language scale, “Social Cognitive Measures Related to Adolescent Dietary Behaviors,” validated by Dewar et al. in 2012 [12]. The Dewar scale was developed based on a literature review and evaluated qualitatively by four experts and ten adolescents. It included 37 items organized into seven scales, five from the personal sphere—self-efficacy, intentions (proximal goals), behavioral strategies, outcome expectations, and expectancies (valuation of outcomes)—and two from the socio-environmental sphere: situation (perceived environment) and social support (from parents or guardians) [12]. For the creation of the SQAEB-SCT, the Spanish translation and cultural adaptation were validated, and five experts and 15 adolescents qualitatively reviewed the scale. The SQAEB-SCT assesses six SCT constructs related to food, grouped into three factors or sections (according to exploratory factor analysis—EFA). 1. Social–environmental constructs: “facilitators (at home) and social support (from parents or guardians)”, 2. personal constructs: “self-efficacy and self-regulation”, and 3. personal constructs in relation to the results: “expectations/valuation of outcomes”. It consists of 22 items, with a Likert-type response format. Its internal consistency was good (Cronbach’s alpha = 0.824), and its temporal stability was excellent (Intraclass Correlation Coefficient—ICC = 0.849) [11]. One limitation of this scale was that confirmatory factor analysis (CFA) was not performed to corroborate the model fit identified in the EFA. Another limitation related to the scale’s content was the omission of peer social support (only parental support was included) and the lack of assessment of learning models among adolescents (parents, teachers, and peers), which are relevant constructs in this theory. By observing models and demonstrating skills and strategies, people gain information and motivation to act [9]. In the school setting, peers are important role models for students and can be a source of social support. Furthermore, the perceived relatedness to peers can increase adolescents’ self-efficacy and motivation to engage in healthy behaviors [9].

To our knowledge, no other SCT scales applied to eating behavior have been developed and validated in Spanish for adolescents. Only specific scales to assess “self-efficacy” in eating among adolescents/adults [13] and among young people [14], validated in the Mexican population, were found.

The objective of this study was to develop a new version based on the review of the SQAEB-SCT, called the “SCT-based scale for Factors Influencing Eating Behavior in Adolescents (SCT-FIEBA)”, and evaluate its reliability and validity. This version, also in Spanish, contains more constructs and items (initially *n* = 35) than the first version. It includes four personal constructs (the same as in the SQAEB-SCT) and four socio-environmental factors applied to eating behavior, with the addition of learning models and peer social support (Supplementary Material S1). Construct validity was analyzed via exploratory factor analysis (EFA) and confirmatory factor analysis (CFA). The psychometric properties evaluated included internal consistency and temporal stability (test–retest). Additionally, analyses were conducted to identify patterns influencing eating behavior based on the SCT; it is worth noting that this analysis had not been performed in the SQAEB-SCT.

## 2. Materials and Methods

### 2.1. Scale Development

The SCT-FIEBA was developed in collaboration with three experts who hold a master’s or doctoral degree and have at least five years of professional experience in nutrition or health education. Based on a review of the previous version (SQAEB-SCT) and the SCT literature, the scale’s new sections and items were developed by experts. Subsequently, three other experts, as well as six adolescents (high school students, four women and two men, aged 15 to 17), qualitatively reviewed the scale using open-ended questions to assess the items’ clarity and relevance (for example: Is the wording of the questions clear? Do you consider any of the questions unrelated to the rest of the questions?). The sample of experts and adolescents was chosen for convenience. The necessary modifications were made to the wording of the items. No item required removal in this qualitative evaluation. No quantitative content evaluation procedures were carried out.

Two sections on key constructs of the SCT were added: “peer social support,” which includes four questions about how often friends or classmates support healthy eating habits, and “learning models,” which includes six questions about the degree of agreement with the representation of learning models of healthy eating habits, including parents or guardians, teachers or instructors, and friends or classmates. Three additional questions were included for constructs already present in the scale’s first version (one on self-efficacy and two on outcome expectations), bringing the total to 35 items. Each item uses a Likert-type response format, with 4 to 6 response options (Supplementary Material S1). Composite scores for the whole scale and its subscales were obtained by aggregating the individual item scores.

### 2.2. Participants

The study included high school students (from all grades, 1st to 3rd years) from the Guadalajara Metropolitan Area, Jalisco, Mexico. Males and females were included, belonging to middle (14–17 years) or late (18–22 years) adolescence [1,15]. The surveys were administered between January and March 2022.

The recommended sample size for survey validation is at least ten participants per item [10,16]; therefore, 350 participants were required. On the other hand, a sample size of ≥200 participants was considered acceptable, ≥300 good, and ≥500 very good [10,16]. The sample size for this study was based on these recommendations (no power analysis was performed), so the sample consisted of 705 participants. For the factor analysis, the sample was randomly divided into two subsamples (using SPSS, Select cases/Random sample of cases/352 of the first 705 cases): one subsample for the EFA (*n* = 353) and the other for the CFA (*n* = 352). Subsequently, internal consistency and pattern identification analyses were performed with the full sample, as well as test–retest reproducibility (43 participants completed the retest).

The participating high schools (*n* = 2) were randomly selected (through Google’s random number generator) from among the 27 public high schools of the University of Guadalajara within the Guadalajara Metropolitan Area. Within each high school, 12 participating class groups were selected using the same random method and stratified selection (four groups per grade, two from the morning shift and two from the afternoon shift). All students enrolled in these groups who agreed to participate were included. The exclusion criteria were students with visual or cognitive disabilities; however, none of these situations were present. No incomplete surveys were submitted either.

### 2.3. Scale Administration

This study primarily employed a cross-sectional design to evaluate the psychometric properties of the scale [17]. However, the assessment of temporal stability (test–retest reliability) included a longitudinal component, using repeated measurements from a subsample of participants.

The study was explained to the adolescents, and their doubts were resolved; those who agreed to participate completed the informed consent/assent form and the corresponding scales in digital format (on computers within the high school facilities). The scale was completed in a supervised classroom setting; research staff were present to answer any questions about it. Two weeks after the first administration, a convenience subsample of participants completed the scale again (due to feasibility issues at the participating school, it was not possible to administer the scale to the whole sample). As feedback, all participants received digital educational materials (via WhatsApp).

Since the study was categorized as minimal risk, and because the participants’ cognitive capacity to provide informed assent (aged 14–17 years) or consent (aged 18–22 years) was assessed, parental or guardian consent was waived for minors. This was in accordance with Guidelines 10 and 17 of the International Ethical Guidelines for Health-Related Research Involving Humans [18], which justify such measures under specific conditions of autonomy and risk. The research protocol was approved by the Research and Ethics Committees of the University Center of Health Sciences, University of Guadalajara (registration number CI-06021).

### 2.4. Analysis

The analyses were performed using SPSS version 27 (IBM, Armonk, NY, USA, 2020) and JASP 0.96 (University of Amsterdam, Amsterdam, The Netherlands, 2026) for the EFA, CFA, and assessment of internal consistency.

To assess construct validity, an EFA was first performed. The analysis was based on the polychoric correlation matrix because the items were ordinal (Likert scales with 4 to 6 options) [16]. The Minimum Residual extraction method (mathematically equivalent to Unweighted Least Squares) was used; this is suitable when using polychoric correlations and when normality is not assumed, and it works well with small or moderate samples for EFA [16]. In this study, although most variables approach normality (kurtosis and skewness between −2 and 2), two outcome expectation items (items 3 and 4) fall outside this range (Supplementary Material S2, Table S1). Promax rotation was applied (oblique rotation) because it allows correlations between factors [19]. For the Kaiser–Meyer–Olkin (KMO) test, values > 0.7 were considered acceptable and >0.8 satisfactory [16]. For Bartlett’s test, a *p*-value < 0.05 was sought [20]. Factor loadings ≥ 0.40 were considered acceptable and ≥0.50 ideal [20].

A CFA was subsequently performed (with a different subsample from the EFA) [16], using the Weighted Least Squares with Mean and Variance adjustment (WLSMV) extraction method. WLSMV is a suitable method for categorical data (or Likert-type scales with fewer than 6 response options) or data that do not follow a normal distribution, and it also works well with samples of 200 or more [21]. Minimum factor loadings of 0.50 were considered adequate; for the root mean square error of approximation (RMSEA) and standardized root mean square residual (SRMR), values < 0.05 indicated a good fit to the data and values between 0.05 and 0.08 a reasonable or acceptable fit [22]. For the incremental fit measures, CFI (Comparative Fit Index) and TLI (Tucker–Lewis Index), values ≥ 0.90 were considered as good fit, and values from 0.80 to 0.89 marginal but acceptable fit; lower values were considered unacceptable (poor fit) [22].

To refine the model, modification indices were examined to detect potential parameters that might affect the overall fit (such as cross-loadings). Likewise, the residual covariance matrix was analyzed to identify remaining associations between items.

To assess temporal stability (test–retest reproducibility), we used the ICC, with absolute agreement between scores, a bidirectional mixed-effects model, and single measures [23,24]. Values < 0.4 are considered low, 0.4–0.59 are considered sufficient or moderate, 0.60–0.74 are considered good, and ≥0.75 are considered excellent [25,26].

To assess internal consistency, Cronbach’s alpha was used (to allow comparison with previous versions) and complemented by McDonald’s omega, which is suitable for ordinal qualitative variables or quantitative variables without a normal distribution; this coefficient, unlike alpha, uses factor loadings [27]. In both cases, standardized coefficients were used. The interpretation was as follows: unacceptable < 0.50, poor 0.50–0.59, questionable 0.60–0.69, acceptable 0.70–0.79, good 0.80–0.89, and excellent ≥ 0.90 [28].

In addition to the validation process, analyses were conducted to identify patterns influencing eating behavior, based on SCT. Individuals were categorized into clusters according to their scores on the SCT constructs. That is, the predictor variables were obtained by adding up the responses of each item within each construct, obtaining additive variables with scores on different scales (for example, 4–24 points for facilitators) (Supplementary Material S2, Table S2). Therefore, for the analyses, they were considered as continuous variables.

1. Hierarchical cluster analysis: This procedure aims to identify clusters or groups of practically homogeneous subjects based on the selected variables, using an algorithm. For the analysis, Ward’s method, Euclidean distance squared, and standardization with Z-scores were used [29]. Prior to the analysis, it was verified that the variables did not exhibit multicollinearity (tolerance > 0.2 and its inverse, the variance inflation factor, <3 were considered adequate) [30]. To determine the maximum number of clusters, the clustering history was examined [29]. It was observed that, starting with the 5-cluster solution, successive unions led to a greater increase in the coefficients. Therefore, it was decided to analyze the 5-, 4-, and 3-cluster solutions.
2. For each of the solutions obtained, an ANOVA test was performed to confirm that there were statistically significant differences (*p* < 0.05) in the SCT constructs’ scores between the groups obtained by each cluster analysis. Although the variables used show slight variations from normality, in ANOVA this has little or no impact on large samples, as is the case here (*n* = 705) [31]. However, Welch’s ANOVA (instead of one-way ANOVA) and the Games–Howell post hoc test were used because the data did not meet the assumption of homogeneity of variances (Levene’s test, *p* < 0.05) [31]. Subsequently, the composition of each cluster (low or high values in each construct) and its consistency with the theory were analyzed. The frequencies of the patterns (cluster sizes) in each solution were also analyzed to ensure they did not differ substantially.
3. Discriminant analysis: This analysis yields a model to predict group or cluster membership based on the variables established as predictors. While this analysis is initially performed on a sample of subjects with known group membership, these functions apply to subjects for whom the predictor variables are available but whose group membership is unknown [32]. To perform the discriminant analysis, each cluster analysis classification was used as the grouping variable, and the additive SCT construct scores were used as independent (predictor) variables. All independent variables were entered together, and the within-group covariance matrix was used. The number of correctly classified cases was analyzed for both the original grouped cases and the cases grouped by leave-one-out cross-validation (each case is classified using functions derived from all other cases). The results were also analyzed using Wilks’ Lambda, the canonical correlation coefficient, and the percentage of variance explained by function 1. Based on these results and the qualitative analysis of the clusters obtained, the solution with the most appropriate number of clusters was chosen.

## 3. Results

### 3.1. Participants Characteristics

The sample comprised 705 high school students, with a mean age of 16.5 years (SD = 1.2; min. 14; max. 22); 559 (79.3%) were in mid-adolescence (14–17 years) and 146 (20.6%) in late adolescence (18–22 years; only one participant was 22 years old). There were 372 women (52.8%) and 333 men (47.2%). The study included 252 (35.8%) first-year students, 247 (35.0%) second-year students, and 206 (29.2%) third-year students. Of these, 377 (53.5%) studied in the morning shift and 328 (46.5%) in the afternoon shift.

Subsample 1 included 353 students with a mean age of 16.6 years (SD = 1.3, min. 14, max. 22); 191 (54.1%) were female, and 162 (45.9%) were male. Subsample 2 included 352 students, with a mean age of 16.5 years (SD = 1.2, min. 14, max. 21); 181 (51.4%) were female, and 171 (48.6%) were male. No significant differences in age and sex were found between these subsamples.

In the retest, 26 females (60.5%) and 17 males (39.5%) participated (mean age: 15.8, SD = 1.1, min. 14, max. 19). There were no significant differences in sex distribution relative to the total sample, but there were differences in age.

### 3.2. Exploratory Factor Analysis

Initially, a parallel analysis was used to determine the number of factors [16], yielding seven factors (KMO test: 0.866; Bartlett’s test, *p* < 0.001). The cumulative variance explained by the seven factors was 63.0%. All variables yielded factor loadings >0.4 on their corresponding factors (between 0.429 and 0.966), without overlap between factors (cross-loading) and communalities > 0.4 (0.490–0.922). The variables of parental social support and self-regulation were combined into a single factor.

The analysis was also performed using eight factors (because the original structure of the scale, from a theoretical perspective, consists of eight constructs), and the results were as follows: KMO = 0.866, Bartlett’s test, *p* < 0.001, and cumulative variance = 65.0%. All variables yielded factor loadings > 0.4 on their corresponding factors (ranging from 0.427 to 0.896) and communalities between 0.495 and 0.884. Only item 1 of the learning models showed cross-loadings on other constructs, specifically parental social support, with very similar loadings of 0.427 and 0.484, respectively. This item concerns whether parents/guardians consume healthy foods (e.g., fruits and vegetables); therefore, it is theoretically related to parental social support. Furthermore, to evaluate the need for item removal, each of the loadings was squared and the ratio between the highest and lowest loading was calculated, resulting in 1.3, which represents a problematic cross-loading [20]. Consequently, it was decided to remove it from the instrument.

The eight-factor analysis was performed again with 34 items, removing the cross-loading item (Table 1). The results were as follows: KMO = 0.870, Bartlett’s test, *p* < 0.001, and cumulative variance = 65.3%. All variables yielded factor loadings >0.4 on their corresponding factors (0.467–0.969) and communalities between 0.475 and 0.885.

### 3.3. Confirmatory Factor Analysis

According to the EFA, two models were tested:

Model 1: Structure with seven factors and 35 items (with self-regulation and parental social support grouped into a single factor).

Model 2A: Structure with eight factors (one for each SCT construct) and 34 items (elimination of item 1 from learning models).

The model fit indices are shown in Table 2. The chi-square *p*-values were significant; however, they were not considered when evaluating the model fit because these values are strongly influenced by sample size.

The seven-factor model showed a reasonable RMSEA (0.079), SRMR above the 0.8 threshold (0.087), and marginal CFI and TLI values (0.89 and 0.88); factor loadings ranged from 0.610 to 0.933. In the eight-factor model with 34 items, the RMSEA absolute fit index indicated a reasonable fit (0.075), the RSMR remained above the expected limit (0.85), and the incremental fit indices indicated a good fit (CFI = 0.90, TLI = 0.90); factor loadings ranged from 0.674 to 0.933. Therefore, this structure was preferred over the seven-factor structure.

Subsequently, it was observed in the modification indices that item 4 of peer support presented high indices (>80) in cross-loadings with five other factors (self-efficacy, self-regulation, learning models, parental social support, and outcome valuation). It was decided to eliminate this item (regarding the consumption of healthy meals or snacks in the company of peers), due to its high multidimensionality and because when analyzed qualitatively, it was observed that it is presented in a very general way and therefore is closely related to other factors.

**Figure 1:**
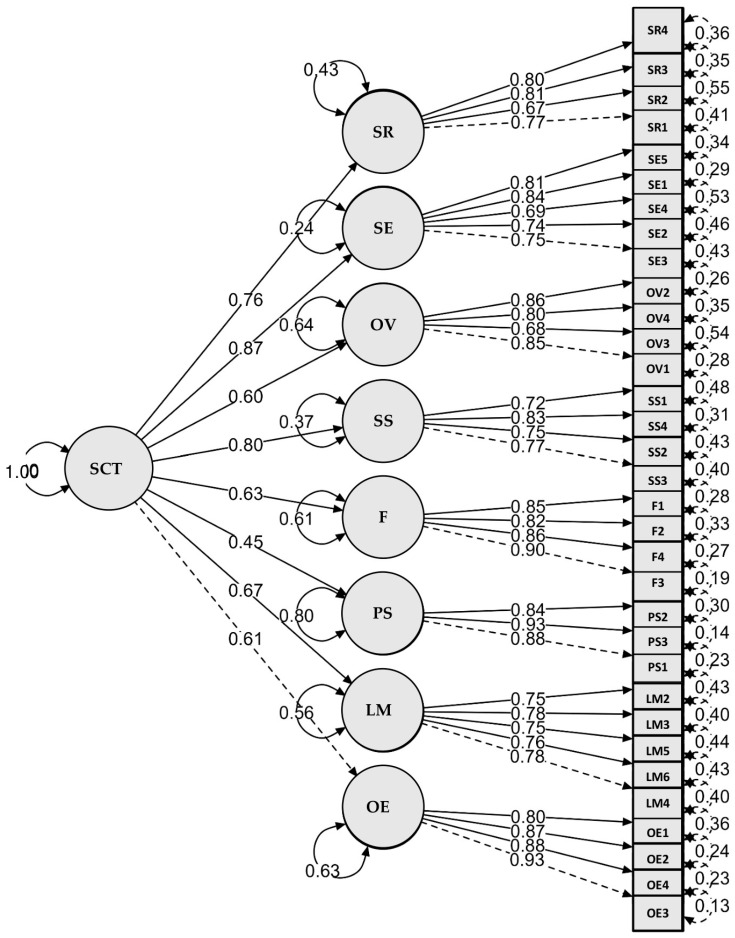
Confirmatory factor analysis with eight factors (33 items) in the SCT-FIEBA (*n* = 352). SCT: Social Cognitive Theory, SR: self-regulation, SE: self-efficacy, OV: outcome valuation, SS: parental social support, F: facilitators, PS: peer social support, LM: learning models, OE: outcome expectations. Note: Dashed arrows represent paths constrained to 1.0 as reference indicators to establish factor scales; solid arrows represent freely estimated parameters. All reported coefficients are standardized.

The analysis was performed (Table 2, Model 2B) with eight factors, with 33 items (without peer social support item 4). In this model, the fit indices improved, indicating a reasonable fit (RMSEA = 0.073) or good fit (CFI = 0.91, TLI = 0.90), although the SRMR remained slightly elevated (0.083). The factor loadings were satisfactory (0.674–0.933) (Figure 1). It was decided to maintain this structure for the SCT-FIEBA, showing acceptable overall fit.

The residual covariance matrix was also analyzed, and it was found that most interactions between items were below 0.20 (raw residuals). The only exception was item 6 of learning models, which showed some residuals exceeding this threshold (0.22, 0.23, and 0.27 in specific interactions). However, it was decided to retain this item in the final structure to preserve the theoretical representation of the model, given that the fit indices were acceptable.

### 3.4. Internal Consistency

Table 3 presents the internal consistency analysis for each resulting section of the scale. The values ranged from moderate to excellent for both Cronbach’s alpha (0.807 to 0.900) and McDonald’s omega (0.808 to 0.903), with both statistics yielding similar values for each section. In all sections, the item-total correlation was >0.5 for all items, and the factor loadings were >0.6. For the peer social support construct, “McDonald’s omega if the element is removed” could not be calculated because the construct contains three items and the analysis cannot be carried out with two items; however, this did not affect the interpretation of the internal consistency of the construct, since the factor loadings and the omega value were satisfactory.

The overall internal consistency of the whole scale was excellent, with Cronbach’s alpha = 0.924 (CI 0.916–0.933) and McDonald’s omega = 0.925 (CI 0.917–0.932).

### 3.5. Test–Retest Reproducibility

Test–retest reproducibility analyses were performed for each section and for the whole scale (Table 4). The ICC values (single measures) ranged from sufficient or moderate to good for the sections (0.436 to 0.745) and were good for the whole scale (0.695).

### 3.6. Identification of Factor Patterns That Influence Eating Behavior According to SCT

First, multicollinearity among the variables used (scores for each factor) was ruled out; both the tolerance (0.514 to 0.741) and the variance inflation factor (1.350 to 1.945) were within adequate ranges. Hierarchical cluster analysis was performed with three, four, and five clusters (according to the analysis of the agglomeration history). Subsequently, through Welch’s ANOVA analysis, it was confirmed that there are statistically significant differences in the scores of the SCT constructs between the groups yielded by each cluster analysis. In all cases, the statistical significance was *p* < 0.001. When analyzing the cluster distribution, the five-cluster solution showed a cluster with only 4.0% (*n* = 28), so the four- or three-cluster solutions, with more balanced distributions, were preferred.

Table 5 compares the results of the discriminant analysis. All three cluster structures (three, four, and five clusters) yielded satisfactory results. The four-cluster structure was retained because it exhibited a lower Wilks’ Lambda and a higher canonical correlation for function 1 compared to the three-cluster structure. Furthermore, the percentage of variance explained by function 1 was adequate (71.1%) and higher than that of the five-cluster structure (62.1%). The percentages of correctly classified original cases were similar across the three models (87–90%).

In the four-cluster structure, the tests for equality of means (Wilks’ Lambda) showed *p*-values < 0.001 for all variables, confirming differences between the clusters. Box’s M test indicated significant differences (*p* < 0.001) in covariance matrices; however, this test exhibits high sensitivity to larger sample sizes, numbers of clusters, and numbers of independent variables, tending to reject the assumption of homogeneity in marginal deviations [33].

The model yielded three significant functions (*p* < 0.001) (Table 6). Function 1 explained 71.1% of the variance, function 2 explained 27.0% of the variance, and function 3 explained 1.9%. According to the standardized canonical structure matrix, for function 1, the variables with the highest loadings in this categorization were greater self-efficacy, self-regulation, parental social support, and learning models. Function 2, on the other hand, considered mainly lower outcome expectations, and function 3 primarily considered greater peer social support to classify the students.

According to the group centroid functions, function 1 primarily distinguished between pattern 2 and pattern 4; therefore, pattern 2 showed higher values and pattern 4 lower values in most constructs. Function 2 mainly distinguished between pattern 1 and pattern 4, so pattern 4 shows markedly lower values in outcome expectancies. Function 3 distinguished between pattern 2 and pattern 3; therefore, pattern 2 has a significantly higher score in peer support than pattern 3.

Table 6 also presents the Fisher functions. These functions generate a linear equation for each pattern using the predictor variable scores. To assign a subject to a pattern, the value of each predictor variable is multiplied by its specific coefficient and the constant is added. The subject is then assigned to the group in which they obtain the highest classification score.

Table 7 describes the characteristics of each cluster (patterns influencing eating behavior based on the SCT) and their frequencies. The percentage of cases correctly classified by the discriminant analysis functions was 86.8%, being higher for pattern 2 “High values in all constructs, especially in peer social support” (96.6%) and lower for pattern 3 “Average values across all SCT constructs, but high in outcome expectations” (83.1%). The results of the cross-validation were very close, with a correct classification in 82.1% of cases, confirming predictive validity.

## 4. Discussion

The SCT-FIEBA showed evidence of reliability and validity in assessing factors influencing adolescent eating behaviors within the SCT framework. Confirmatory factor analysis showed an acceptable fit for the eight-factor model. Furthermore, the instrument’s reliability and its ability to identify distinctive factor patterns influencing eating behavior suggest that it is a useful tool for exploring the personal and environmental factors that influence eating. The constructs included were, from the personal domain, self-efficacy, self-regulation, outcome expectancies, and outcome valuation; from the socio-environmental domain, they were learning models, parental social support, peer social support, and facilitators. The SCT, through the concept of reciprocal determinism, highlights the importance of considering both types of constructs (personal and socio-environmental) as modifiers of eating behavior and emphasizes that, although they are distinct concepts, they are closely related, and modifying one can lead to changes in the other [9].

The sample primarily included students in mid-adolescence (14–17 years old) (79%), a stage characterized by a search for independence, concern for physical appearance, and peer acceptance. They exhibit a greater capacity for complex decision-making; however, they still tend to act on impulse or emotion [15]. Therefore, it is a stage in which both personal and socio-environmental factors are of great importance. On the other hand, 21% were in the late adolescent stage, from 18 to 22 years old (those over 19 years old were likely included due to delayed schooling or grade repetition), a stage in which they tend to have greater impulse control and the ability to weigh risks versus benefits. However, brain development is not complete until around age twenty-five [1,15]. It should be noted that sensitivity analyses were performed excluding late adolescents, and it was confirmed that the factor structure and psychometric measures of the scale did not change significantly.

To assess construct validity, an EFA was initially performed on a subsample of 353 adolescents. The seven- and eight-factor solutions were satisfactory, although the eight-factor solution provided the most interpretable factor structure (one factor for each construct of the theory). The cumulative variance explained was 63.0% and 65.3%, respectively (KMO = 0.87; Bartlett’s test, *p* < 0.001). In the seven-factor structure, two constructs were grouped into a single factor (self-regulation and parental social support), likely due to their close relationships within SCT. During adolescence, parental social support significantly influences personal aspects, such as self-efficacy and self-regulation [34]. However, while related, they measure different aspects. The items on parental social support ask whether parents prepared healthy snacks and meals for the adolescent and encouraged them to eat healthy, while the self-regulation item asks whether the adolescent chose and prepared healthy foods. Only item 4 on parental social support shows a stronger theoretical relationship, as it asks whether the adolescent prepared healthy meals or snacks with their parents.

In the initial eight-factor structure (35 items), the presence of a cross-loading item is also due to the close relationship between the constructs; item 1, on learning models, also showed a loading > 0.4 on parental social support, since both constructs refer to the influence of parents/guardians on adolescent eating. The questions for the first construct explore whether parents are healthy-eating role models, and those for the second explore how they support and motivate adolescents to eat healthily. However, it was decided to eliminate this item, considering the closeness of the factor loadings, a better model fit by removing the item, and the fact that there is no significant loss of construct coverage (one item on parents as role models, two on teachers, and two on peers are maintained).

The CFA, performed on an independent subsample (*n* = 352), showed that the eight-factor structure (34 items) provided a better model fit compared to the seven-factor model. However, the modification indices (cross-loadings) indicated that item 4 of peer social support was highly correlated with other factors and was not specific to the construct for which it was designed, thereby affecting the model’s fit. Therefore, it was decided to remove it without affecting the representation of the construct; furthermore, the CFA showed that the model fit improved after its removal. Acceptable fit indices were obtained (CFI = 0.91, TLI = 0.90, RMSEA = 0.073), with the exception of the SRMR index (0.083) which remained slightly above the conventional threshold.

All variables showed adequate factor loadings (>0.60), supporting the model’s structural stability.

In the previous version of the scale (SQAEB-SCT), with a sample of 152 adolescents [11], only EFA was performed, obtaining satisfactory results with three factors (grouping of two SCT constructs for each factor) with KMO = 0.80, Bartlett’s test *p* < 0.001, loadings > 0.39, and cumulative variance percentage of 41.9%. Compared with the SQAEB-SCT, the SCT-FIEBA did not group outcome expectation items with outcome valuation items, nor did it group facilitator items with parental social support items.

In contrast, the Dewar scale [12] on “Social Cognitive Measures Related to Adolescent Dietary Behaviors” (in English) (*n* = 173), on which the SQAEB-SCT was originally based, used CFA for each section independently (using single-factor models), with satisfactory model fit indices (CFI = 0.94 to 1.00; RMSEA = 0.00 to 0.08 and only one construct with 0.11) and loadings mostly greater than 0.40 (except the outcome valuation construct, which had loadings from 0.23). Another scale based on SCT to assess the physical activity behavior of Iranian adolescent girls (SCT-PAIAGS) [35] was validated (in Persian-language) in 400 adolescent women. Once the validity of the content had been verified, CFA was performed with six factors, one factor for each SCT construct included: self-efficacy, self-regulation, family support, friend support, outcome expectancy, and self-efficacy to overcoming impediment; a goodness of fit index (GFI) of 0.84, RMSEA of 0.04, SRMR of 0.02, and factor loadings between 0.11 and 0.62 were obtained, with significant t values (*p* < 0.05), which indicated the significance of the factor loadings.

Another study, with results similar to the present study, was conducted with 1375 preadolescents (10–12 years old) in China. The “Children’s Eating and Physical Activity Behavior Questionnaire (CEPAB-Q)”, based on SCT [36], was validated. This was adapted and translated into Chinese from a previous scale (Food, Health, and Choice Questionnaire). Fifty-five items on self-determination, outcome expectations, self-efficacy, habit strength, and goal intention were included, all of which demonstrated satisfactory content validity. The five-factor CFA (one factor for each construct) was not satisfactory, so four items (with loadings < 0.4) on outcome expectations were removed, and the structure was modified to six factors. The outcome expectations construct was divided into two factors: one related to the benefits of “do more” (consuming healthy foods and physical activity), and the other to the benefits of “do less” (consuming unhealthy foods and screen time). The model fit with this structure ranged from acceptable to very good (CFI = 0.91, TLI = 0.90, RMSEA = 0.05, SRMR = 0.06) [36].

Regarding reliability, the SCT-FIEBA was evaluated for test–retest reproducibility, yielding acceptable values despite the smaller sample size (*n* = 43). The ICC was good for four of the sections (parental social support, learning models, self-regulation, and self-efficacy) and the whole scale (ICC = 0.695). The other four sections (facilitators, peer social support, outcome expectations, and valuation) presented moderate values. No section showed low values (<0.40), but none showed excellent values (>0.75). The small sample size may have influenced these values. Another factor that can influence the results is the type of construct being measured and its stability over time [10]. In this study, the constructs that showed the greatest reproducibility were those related to the ability to perform healthy behaviors (self-regulation) and the perception of those behaviors (self-efficacy), as well as parental social support. Conversely, the construct with the least reproducibility (outcome expectations) refers to beliefs about the benefits of healthy behaviors, an aspect that may be more unstable over time, followed by peer social support. This had already been observed during validation of the SQAEB-SCT (*n* = 70): the self-efficacy/self-regulation factor showed the highest ICC, and the outcome expectations/outcome valuation factor showed the lowest ICC [11]. Some similarities were also observed in the validation of the SCT-PAIAGS: the self-efficacy factor showed the highest ICC (0.90), and the peer support factor showed the lowest ICC (0.73) [35].

It is worth highlighting the psychometric rigor employed in the analysis: a bidirectional mixed-effects model was used, and the absolute agreement between scores and individual measures (which yields lower values than using the ICC for consistency and the mean of k measures) was considered. This is the most appropriate form of the ICC in test–retest studies [23,24]. However, a lack of reporting on the ICC methods used in research has been observed, which can lead to incorrect application of the ICC form [23].

Similarly, the scale items were consistent with one another in assessing the SCT constructs, with Cronbach’s alpha values ranging from moderate to excellent for the sections (0.807 to 0.900) and excellent for the whole scale (0.924). The results, using McDonald’s omega statistic, were very similar, confirming the internal consistency of the scale and its sections. Qualitative analysis ruled out the possibility that sections with coefficients close to or greater than 0.9 were redundant. For example, the “outcome expectations” section assesses the perception of different benefits of healthy eating: reduced risk of disease, improved concentration at school, weight control, and increased energy levels.

These values were similar to the SQAEB-SCT [11], which showed excellent reproducibility, with an ICC of 0.849 for the whole scale (ranging from 0.606 to 0.882 for the sections) and good internal consistency, with a Cronbach’s alpha of 0.824 for the scale (ranging from 0.61 to 0.82 for the sections). Meanwhile, Dewar’s study also showed good results for reproducibility (ICC of 0.81 to 0.89) and internal consistency (Cronbach’s alpha of 0.65 to 0.79) [12]. Similarly, satisfactory results were obtained in the SCT-PAIAGS; the ICC ranged from 0.73 to 0.90, and Cronbach’s alpha ranged from 0.78 to 0.85 across the scale’s sections [35]. Internal consistency was also adequate in the CEPAB-Q, with a Cronbach’s alpha of 0.95 for the scale and 0.85–0.92 for the sections, except for one section with a value of 0.66 (outcome expectations “do more”) [36].

Another study investigated the impact of the SCT constructs on healthy eating habits among adolescents in Thailand [37]. The researchers developed a 54-item scale (language not specified) with three- to six-point Likert-type responses. This scale assessed self-efficacy, intentions, outcome expectations, perceived benefits, perceived barriers, situations, parental/guardian social support, and peer social support. The scale demonstrated content validity (three experts) using the Index of Item-Objective Congruence (average scores > 0.5) and adequate internal consistency (Cronbach’s alpha) for the whole scale (0.81) and for the sections (0.66 to 0.85). However, the instrument’s reproducibility and construct validity were not assessed [37].

Additionally, this study evaluated the usefulness of the SCT-FIEBA to identify psychosocial patterns that influence eating behavior. The identification of four distinct patterns (via cluster analysis) yielded good results in Welch’s ANOVA and equality-of-means tests in discriminant analysis (*p* < 0.001 for all variables); that is, adolescents across the patterns showed significantly different scores on each SCT construct. In addition, the percentage of correctly classified cases was 87%, indicating that the functions obtained are useful for adequately classifying participants into different patterns. Furthermore, the results of this analysis are supported by cross-validation, which also showed good classification accuracy (86.2%), demonstrating the model’s stability and the absence of critical overfitting.

The usefulness of these patterns lies in a deeper analysis of the factors influencing adolescents’ eating behavior in relation to SCT constructs. For example, in this study’s population, 31.5% of participants were classified in pattern 1, with low values across most constructs, except for outcome expectations and facilitators, which had average values; therefore, in these adolescents, all constructs need to be reinforced. The most frequent pattern, pattern 3 (43.7%), was characterized by average values across all SCT constructs but was high in outcome expectations. In contrast, participants in pattern 4 (8.2%) showed low values across most SCT constructs, particularly in outcome expectations, suggesting that this construct requires further development in these adolescents. This analysis could be useful for planning nutritional interventions with a psychosocial component that addresses the specific needs of an individual or a school group.

It should be mentioned that none of the scales mentioned above assessed the identification of patterns influencing eating behavior according to SCT. To our knowledge, this type of analysis has been carried out previously on questionnaires or scales assessing the quality of food consumption, but has not been carried out on psychosocial constructs related to eating in adolescents. In the study by Bernal-Orozco [38] in Mexico, the reproducibility and pattern identification capacity of the “Mini-Survey to Evaluate Food Intake Quality Version 2 (Mini-ECCA v2)” were validated in 276 university students. Moderate-to-excellent reproducibility was found (weighted kappa between 0.422 and 0.662), and three food consumption patterns were identified through cluster analysis. Differences between groups were confirmed with ANOVA (*p* < 0.05), and discriminant analysis showed satisfactory results (eigenvalue of function one: 2.594, canonical correlation close to one: 0.849, Wilks’ Lambda close to zero: 0.213, and chi-square with *p* < 0.001). The obtained functions correctly classified 85.9% of cases. Likewise, in another study involving 83 Native American adults [39], four patterns of factors influencing food selection were identified through a questionnaire. Cluster analysis was used, and the differences between the patterns were subsequently confirmed with MANOVA (*p* < 0.001) and discriminant analysis (Wilks’ Lambda of 0.048); 97.3% of the cases were correctly classified. The results in these studies are similar to those of the present study.

### Limitations and Strengths

It should be noted that this study has some limitations. As this is a self-report study, adolescents’ responses may be subject to bias. However, participants were informed about the importance of thoughtful and honest answers, and researchers were present during scale completion to address any questions.

Regarding the constructs included in the scale, one limitation is that it considered only facilitators at home, not in the school environment (availability of healthy food, and marketing and advertisements in and around the school). For future similar studies, it is recommended that questions about school facilitators be included. Furthermore, in future scales, current digital influences within socio-environmental constructs could be considered, for example, the role of social media content creators as learning models in eating.

Another limitation is the lack of quantitative validation of the scale’s content by an expert panel, which would strengthen evidence for its content validity. Although experts and adolescents participated in developing the different versions of the scale, the methods used were solely qualitative; no quantitative content validity index or structured rating procedure was used. Comprehensive content validation, including quantitative aspects, is an important component of scale development.

In factor analysis, the removal of two items represents a potential limitation, as eliminating items based primarily on empirical criteria could reduce the construct’s conceptual coverage. However, qualitative analyses were conducted to ensure that the representativeness of the constructs (learning models and peer social support) and the whole scale was not affected by the item removal.

Regarding the sample size for the test–retest reliability analysis (*n* = 43), this was relatively small and selected by convenience sampling (due to feasibility constraints at the participating school), which could limit the precision of the stability estimates and the representativeness of the population (for example, this subsample had a lower average age compared to the total sample). These issues may limit the strength of the evidence for temporal stability. While a sample size of at least 30 participants is recommended for calculating the ICC [23], it is advisable to corroborate these results in larger, more diverse cohorts to ensure the instrument’s reproducibility. In this study, four constructs showed barely sufficient ICCs (facilitators, peer social support, outcome expectations, and outcome valuation), with values of 0.44–0.56, and very wide values in the 95% CI, showing limited statistical precision, probably due to the small sample size. Therefore, the reproducibility of these constructs should be approached with caution and corroborated.

Finally, given that the scale was validated in Spanish, in Mexican adolescents who were students in an urban area, its use in populations with different characteristics (for example, in rural areas or non-school-attending adolescents), adolescents from other Spanish-speaking countries, or those with a different language could require sociocultural linguistic adaptation and/or the validation of its translation into other languages, as well as a reassessment of the psychometric characteristics of the scale in that population.

Additional studies are warranted to confirm that the revised scale maintains adequate content validity and generalizability across different populations.

Nevertheless, the present study is strengthened by the SCT-FIEBA’s reliability in assessing eight SCT constructs applied to adolescents’ eating behavior, either independently or in groups, as each section and the whole scale showed satisfactory results. Another strength of the study is the scale’s ability to identify patterns influencing eating behavior. As a result, the interpretation of the scale goes beyond a score, allowing for an understanding of how the different SCT constructs manifest and interact in each evaluated population or individual.

Furthermore, it is important to consider that few validation studies of scales based on Theories for Behavior Change have assessed the factors that influence eating behavior in adolescents. To our knowledge, no other SCT scales applied to eating behavior have been validated in Spanish. The SCT-FIEBA has the advantage of assessing the most representative learning models for adolescents: parents, teachers, and peers (friends/classmates), as well as peer social support, which plays a crucial role during adolescence.

Another strength of this study is the practical application of this scale. At the clinical or individual level, it will enable a more comprehensive assessment and more personalized interventions in adolescent care. At the population level, its use in a nutritional assessment can facilitate the planning and implementation of educational interventions and public health programs for adolescents. At the research level, it is proposed to evaluate its usefulness in predicting health outcomes, such as diet quality, eating disorders, or overweight/obesity. However, it is highly recommended to use the SCT-FIEBA alongside validated adolescent surveys that provide quantitative data on food consumption, eating patterns, and eating practices (such as mealtimes, meal locations, and company at meals) to achieve a more complete assessment of eating behavior and associated factors.

Given the proven usefulness of SCT in various health promotion initiatives, the development, adaptation, and validation of SCT-based scales for diverse populations are essential. This study revised and validated the SCT-FIEBA, designed for Spanish-speaking adolescents, with good results. Its use in clinical, population, or research settings can be helpful for describing, analyzing, and addressing the personal and socio-environmental factors that influence adolescent eating behavior.

## 5. Conclusions

The SCT-FIEBA showed evidence of validity and reliability for assessing SCT constructs applied to eating behavior in high school adolescents, in a Mexican population, as well as for identifying four distinct psychosocial patterns that influence eating behavior. However, it is recommended to corroborate the instrument’s temporal stability in a larger sample. The application of the SCT-FIEBA, in conjunction with other instruments, can contribute to a more comprehensive assessment of adolescent eating and the factors that influence it.

## Figures and Tables

**Table 1:** Exploratory factor analysis of eight factors (34 items) in the SCT-FIEBA (*n* = 353).

Items	Factors								Communalities
	1	2	3	4	5	6	7	8	
Outcome Expectations 1	0.778								0.636
Outcome Expectations 2	0.780								0.662
Outcome Expectations 3	0.900								0.866
Outcome Expectations 4	0.870								0.885
Peer Social Support 1		0.894							0.790
Peer Social Support 2		0.902							0.750
Peer Social Support 3		0.902							0.805
Peer Social Support 4		0.546							0.515
Facilitators 1			0.672						0.733
Facilitators 2			0.703						0.611
Facilitators 3			0.969						0.878
Facilitators 4			0.816						0.771
Learning Models 2				0.578					0.475
Learning Models 3				0.700					0.571
Learning Models 4				0.895					0.728
Learning Models 5				0.693					0.631
Learning Models 6				0.767					0.696
Outcome Valuation 1					0.813				0.762
Outcome Valuation 2					0.773				0.730
Outcome Valuation 3					0.787				0.607
Outcome Valuation 4					0.768				0.564
Self-efficacy 1						0.539			0.564
Self-efficacy 2						0.723			0.599
Self-efficacy 3						0.855			0.705
Self-efficacy 4						0.707			0.495
Self-efficacy 5						0.532			0.573
Parental Social Support 1							0.467		0.526
Parental Social Support 2							0.749		0.623
Parental Social Support 3							0.745		0.602
Parental Social Support 4							0.634		0.638
Self-regulation 1								0.718	0.517
Self-regulation 2								0.689	0.488
Self-regulation 3								0.534	0.601
Self-regulation 4								0.532	0.600
**Percentage of variance explained by the factor**	9.6	8.6	8.6	8.4	8.0	7.9	7.5	6.7	

Note: Factor loadings ≥ 0.40 are shown; the largest factor loading is highlighted in blue.

**Table 2:** Adjustment indices in the confirmatory factor analysis in the SCT-FIEBA (*n* = 352).

Model	X^2^ (DF), *p* Value	SRMR	RMSEA (90% CI)	CFI	TLI
1. Seven factors (35 items)	1761 (553), <0.001	0.087	0.079 (0.075–0.083)	0.891	0.883
2A. Eight factors (34 items)	1542 (519), <0.001	0.085	0.075 (0.071–0.079)	0.904	0.896
2B. Eight factors (33 items)	1405 (487), <0.001	0.083	0.073 (0.069–0.078)	0.912	0.904

X^2^: chi-square; DF: Degrees of Freedom; SRMR: standardized root mean square residual; RMSEA: root mean square error of approximation; CI: confidence interval; CFI: Comparative Fit Index; TLI: Tucker–Lewis Index.

**Table 3:** Internal consistency of the SCT-FIEBA (*n* = 705).

Construct	Question	Item-Total Correlation	Cronbach’s Alpha if the Element is Removed	Cronbach’s Alpha by Section (95% CI)	Factor Loading	McDonald’s Omega if the Element is Removed	McDonald’s Omega (95% CI)
Facilitators	1	0.748	0.880	0.899 (0.883–0.916)	0.792	0.882	0.900 (0.888–0.912)
	2	0.738	0.884		0.774	0.886	
	3	0.824	0.853		0.897	0.853	
	4	0.796	0.863		0.862	0.864	
Parental social support	1	0.630	0.770	0.815 (0.790–0.840)	0.721	0.769	0.816 (0.794–0.838)
	2	0.667	0.752		0.767	0.755	
	3	0.599	0.785		0.678	0.783	
	4	0.643	0.764		0.732	0.765	
Peer social support	1	0.771	0.818	0.877 (0.856–0.898)	0.853	NA	0.877 (0.861–0.893)
	2	0.748	0.839		0.817	NA	
	3	0.767	0.821		0.847	NA	
Learning models	2	0.561	0.828	0.839 (0.822–0.859)	0.618	0.826	0.840 (0.822–0.859)
	3	0.632	0.807		0.713	0.810	
	4	0.685	0.793		0.764	0.795	
	5	0.649	0.806		0.727	0.808	
	6	0.680	0.796		0.752	0.802	
Self-regulation	1	0.617	0.761	0.807 (0.785–0.831)	0.693	0.767	0.808 (0.785–0.831)
	2	0.567	0.783		0.634	0.786	
	3	0.678	0.733		0.796	0.733	
	4	0.631	0.755		0.737	0.755	
Self-efficacy	1	0.677	0.804	0.844 (0.823–0.865)	0.761	0.805	0.844 (0.826–0.862)
	2	0.644	0.813		0.720	0.813	
	3	0.673	0.805		0.741	0.807	
	4	0.605	0.824		0.662	0.825	
	5	0.646	0.812		0.719	0.814	
Outcome expectations	1	0.690	0.902	0.900 (0.879–0.922)	0.729	0.903	0.903 (0.891–0.914)
	2	0.751	0.879		0.796	0.885	
	3	0.851	0.843		0.926	0.846	
	4	0.810	0.857		0.883	0.861	
Outcome valuation	1	0.673	0.763	0.822 (0.792–0.852)	0.769	0.765	0.823 (0.802–0.844)
	2	0.665	0.766		0.765	0.767	
	3	0.596	0.799		0.667	0.799	
	4	0.642	0.776		0.730	0.779	

CI: confidence interval; NA: not applicable (the analysis cannot be conducted with two items).

**Table 4:** Test–retest reproducibility of the SCT-FIEBA (*n* = 43).

Section	Test–Retest Reproducibility		
	ICC	95% CI	*p*-Value
Facilitators (4 items)	0.467	0.193–0.672	0.001
Parental Social Support (4 items)	0.671	0.467–0.807	<0.001
Peer Social Support (3 items)	0.464	0.201–0.667	<0.001
Learning Models (5 items)	0.625	0.404–0.777	<0.001
Self-regulation (4 items)	0.745	0.575–0.853	<0.001
Self-efficacy (5 items)	0.630	0.413–0.780	<0.001
Outcome Expectations (4 items)	0.436	0.168–0.646	0.001
Outcome Valuation (4 items)	0.555	0.308–0.732	<0.001
SCT-FIEBA (33 items)	0.695	0.503–0.822	<0.001

ICC: Intraclass Correlation Coefficient (bidirectional mixed-effects model, absolute agreement between scores, single measures); CI: confidence interval.

**Table 5:** Results of discriminant analysis with 3, 4, and 5 clusters in the SCT-FIEBA (*n* = 705).

Number of Clusters/Patterns	Wilks’ Lambda	X^2^ (DF), *p* Value	Function 1			Cases Correctly Classified	
			Canonical Correlation Coefficient	Eigenvalue	% Variance Explained	Original *	Cross-Validation +
3	0.179	1200 (16), <0.001	0.825	2.132	73.2	90.1	88.9
4	0.108	1552 (24), <0.001	0.866	2.999	71.1	86.8	86.2
5	0.066	1896 (32), <0.001	0.867	3.033	62.1	87.1	86.8

X^2^: chi-square; DF: Degrees of Freedom. * Original grouped cases are correctly classified with the functions of discriminant analysis; + each case is classified using the functions derived from all cases other than that case.

**Table 6:** Discriminant analysis with four clusters in the SCT-FIEBA (*n* = 705).

Variable (Construct)	Fisher Functions (Classification Function Coefficients)				Standardized Canonical Structure Matrix (Discriminant Loadings)		
	Pattern Influencing Eating Behavior Based on the SCT				Function 1	Function 2	Function 3
	1	2	3	4			
Facilitators	0.547	0.758 +	0.623	0.357 °	0.374 *	−0.100	0.039
Self-efficacy	0.370	0.711 +	0.589	0.363 °	0.514 *	0.181	−0.412
Self-regulation	0.644 °	1.173 +	0.898	0.702	0.411 *	0.358	−0.293
Outcome Expectations	1.543	1.633 +	1.590	0.023 °	0.586	−0.697 *	−0.157
Outcome Valuation	1.670 °	1.878 +	1.891	2.581	0.236	0.152	−0.515 *
Learning Models	0.644	1.007 +	0.771	0.547 °	0.414 *	0.244	0.378
Parental Social Support	0.724	1.198 +	1.031	0.732 °	0.470 *	0.295	−0.335
Peer Social Support	0.835 °	1.533 +	1.050	0.961	0.284	0.411	0.647 *
Constant	−49.556	−92.576	−69.627	−37.636			

+ The higher value (highlighted in green) indicates that the highest scores for that variable are found in that cluster; ° the lower value (highlighted in orange) indicates that the lowest scores for that variable are found in that cluster (Welch ANOVA test, Games–Howell post hoc). * Highest absolute correlation between the variable and the discriminant function.

**Table 7:** Description of the four patterns and correctly classified cases in the SCT-FIEBA (*n* = 705).

Pattern Influencing Eating Behavior Based on the SCT	Description	With Hierarchical Cluster *n* (% of Total)	With the Functions of Discriminant Analysis (% of Total)	Cases Correctly Classified * n (% Within the Cluster)	Cases Correctly Classified in Cross-Validation + *n* (% Within the Cluster)
1	Low values in most constructs, except outcome expectations and facilitators, with average values.	222 (31.5)	223 (31.6)	191 (86.0)	190 (85.6)
2	High values in all constructs, especially in peer social support.	117 (16.6)	136 (19.3)	113 (96.6)	113 (96.6)
3	Average values across all SCT constructs, but high in outcome expectations.	308 (43.7)	288 (40.9)	256 (83.1)	253 (82.1)
4	Low values in most SCT constructs, especially in outcome expectations (average values in peer social support).	58 (8.2)	58 (8.2)	52 (89.7)	52 (89.7)
Total		705 (100)	705 (100)	612 (86.8)	608 (86.2)

* Original grouped cases were correctly classified with the functions of discriminant analysis; + each case is classified using the functions derived from all cases other than that case.

## Data Availability

The data presented in this study are available upon request from the corresponding author, as they are part of an ongoing study.

## Supplementary Materials

The following supporting information can be downloaded at: https://www.mdpi.com/article/10.3390/healthcare14142229/s1, Material S1: Social Cognitive Theory-based scale for Factors Influencing Eating Behavior in Adolescents (SCT-FIEBA). Original version in Spanish and English translation; Material S2: Characteristics of the items and additive variables in the Social Cognitive Theory-based scale for Factors Influencing Eating Behavior in Adolescents (SCT-FIEBA); Table S1: Characteristics and measures of skewness and kurtosis of the items in the SCT-FIEBA (*n* = 705); Table S2: Characteristics and measures of skewness and kurtosis of the additive variables in the SCT-FIEBA (*n* = 705).

## Abbreviations

The following abbreviations are used in this manuscript:

CI	Confidence Interval
SCT	Social Cognitive Theory
SQAEB-SCT	Spanish-language Questionnaire on Eating Behavior in Adolescents based on SCT
SCT-FIEBA	SCT-based scale for Factors Influencing Eating Behavior in Adolescents
EFA	Exploratory Factor Analysis
CFA	Confirmatory Factor Analysis
KMO	Kaiser–Meyer–Olkin
RMSEA	Root Mean Square Error of Approximation
SRMR	Root Mean Squared Residual
CFI	Comparative Fit Index
TLI	Tucker–Lewis Index
ICC	Intraclass Correlation Coefficient
SCT-PAIAGS	Questionnaire-based on SCT to assess the Physical Activity behavior of Iranian Adolescent Girls
CEPAB-Q	Children’s Eating and Physical Activity Behavior Questionnaire
WLSMV	Weighted Least Squares with Mean and Variance adjustment
