# Supplementary material for: Validation of the Revised Version of the Social Cognitive Theory-Based Scale for Factors Influencing Eating Behavior in Adolescents
Source: Healthcare (Basel). 2026 Jul 22;14(14):2229. doi: 10.3390/healthcare14142229 (PMC13411829; doi:10.3390/healthcare14142229)
Supplement: Supplementary file 1 [file healthcare-14-02229-s001.zip › SM 1. 29.06.26.pdf]

SUPPLEMENTARY MATERIAL S1

Social Cognitive Theory - based scale for Factors Influencing Eating Behavior in Adolescents (SCT-FIEBA). Original version in Spanish and English translation.

Note: Items marked with an asterisk were removed in the validated version.

ESCALA DE FACTORES QUE INFLUYEN EN LA CONDUCTA ALIMENTARIA PARA ADOLESCENTES, CON BASE EN LA TEORÍA SOCIAL COGNITIVA

A continuación, te presentamos algunas preguntas relacionadas con tus hábitos alimentarios. Por favor lee las instrucciones de cada sección, reflexiona las preguntas y contesta con sinceridad. Recuerda NO ES UN EXAMEN.

Facilitadores<br><br>Encierra en un círculo el número que indica que tan de acuerdo o desacuerdo estas con cada oración.	1. Muy en desacuerdo	2. En desacuerdo	3. Ligeramente en desacuerdo	4. Ligeramente de acuerdo	5. De acuerdo	6. Muy de acuerdo
1. En mi casa hay refrigerios saludables disponibles para comer	1	2	3	4	5	6
2. En mi casa hay bebidas saludables disponibles (por ejemplo, agua natural, leche, bebidas sin azúcar)	1	2	3	4	5	6
3. En mi casa siempre hay frutas disponibles para comer (incluyendo frescas o secas)	1	2	3	4	5	6
4. En mi casa siempre hay verduras disponibles para comer (ya sea frescas, cocidas o enlatadas)	1	2	3	4	5	6

Apoyo social de Padres<br><br>Encierra en un círculo el número correspondiente para cada pregunta.<br><br>*En el último mes, con qué frecuencia…	1. Nunca	2. Rara vez	3. Algunas veces	4. Frecuentemente	5. Siempre
1. Mis papás (o tutores) prepararon refrigerios saludables para mí (por ejemplo, fruta o yogur sin azúcar)	1	2	3	4	5
2. Mis papás (o tutores) prepararon comidas saludables para mí (incluyendo desayunos, comidas y cenas)	1	2	3	4	5

3. Mis papás (o tutores) me animaron a comer frutas y verduras	1	2	3	4	5
4. Preparé comidas o refrigerios saludables en compañía de mis papás (o tutores)	1	2	3	4	5

Apoyo Social de Pares<br><br>Encierra en un círculo el número correspondiente para cada pregunta.<br><br>*En el último mes, con qué frecuencia…	1. Nunca	2. Rara vez	3. Algunas veces	4. Frecuentemente	5. Siempre
1. Mis amigos o compañeros de clase me animaron a comer frutas y verduras	1	2	3	4	5
2. Mis amigos o compañeros de clase me animaron a tomar agua natural, leche o bebidas sin azúcar	1	2	3	4	5
3. Mis amigos o compañeros de clase me animaron a evitar alimentos altos en grasa o azúcar (frituras, panes, dulces, bebidas azucaradas)	1	2	3	4	5
4. Consumí comidas o refrigerios saludables en compañía de mis amigos o compañeros de clase *	1	2	3	4	5

Modelos de Aprendizaje<br><br>Encierra en un círculo el número que indica que tan de acuerdo o desacuerdo estas con cada oración.	1. Muy en desacuerdo	2. En desacuerdo	3. Ligeramente en desacuerdo	4. Ligeramente de acuerdo	5. De acuerdo	6. Muy de acuerdo
1. Mis papás (o tutores) consumen alimentos saludables (por ejemplo, frutas o verduras) *	1	2	3	4	5	6
2. Mis papás (o tutores) evitan consumir alimentos altos en grasa o azúcar (frituras, panes, dulces, bebidas azucaradas)	1	2	3	4	5	6
3. Mis profesores o instructores consumen alimentos saludables (por ejemplo, frutas o verduras)	1	2	3	4	5	6
4. Mis profesores o instructores evitan consumir alimentos altos en grasa o azúcar (frituras, panes, dulces, bebidas azucaradas)	1	2	3	4	5	6

5. Mis amigos o compañeros de clase consumen alimentos saludables (por ejemplo, frutas o verduras)	1	2	3	4	5	6
6. Mis amigos o compañeros de clase evitan consumir alimentos altos en grasa o azúcar (frituras, panes, dulces, bebidas azucaradas)	1	2	3	4	5	6

Autorregulación<br><br>Encierra en un círculo el número correspondiente para cada pregunta.<br><br>*En el último mes con qué frecuencia…	1. Nunca	2. Rara vez	3. Algunas veces	4. Frecuentemente	5. Siempre
1. Elegí los alimentos con menos grasa o con grasas saludables cuando estos estaban disponibles (por ejemplo: pan tostado en lugar de pan dulce, guacamole en lugar de aderezos)	1	2	3	4	5
2. En lugar de elegir bebidas azucaradas como refrescos o jugos embotellados, elegí agua natural o bebidas sin azúcar (como aguas saborizadas light)	1	2	3	4	5
3. Preparé para mí mismo comidas y refrigerios con ingredientes saludables y bajos en azúcar	1	2	3	4	5
4. Llevé a cabo acciones para hacer las frutas y verduras más agradables (por ejemplo, probar nuevas recetas y combinaciones)	1	2	3	4	5

Autoeficacia<br><br>Encierra en un círculo el número que indica que tan de acuerdo o desacuerdo estas con cada oración.<br><br>*Cuando tengo que elegir un alimento para comer...	1. Muy en desacuerdo	2. En desacuerdo	3. Ligeramente en desacuerdo	4. Ligeramente de acuerdo	5. De acuerdo	6. Muy de acuerdo
1. Me es fácil elegir refrigerios saludables cuando como entre comidas (por ejemplo, fruta o yogur sin azúcar)	1	2	3	4	5	6
2. Creo que tengo los conocimientos y habilidades para elegir y preparar refrigerios saludables	1	2	3	4	5	6
3. Me es fácil elegir comidas/refrigerios saludables cuando salgo a comer con mis amigos	1	2	3	4	5	6

4. Me es fácil evitar alimentos altos en grasa o azúcar (frituras, panes, dulces, bebidas azucaradas)	1	2	3	4	5	6
5. Me es fácil comer frutas y verduras cada día	1	2	3	4	5	6

Encierra en un círculo, el número que indica que tan de acuerdo o desacuerdo estas con cada beneficio y qué tan importante es cada beneficio para ti											
Expectativas de resultados	1. Muy en desacuerdo	2. En desacuerdo	3. Ligeramente en desacuerdo	4. Ligeramente de acuerdo	5. De acuerdo	6. Muy de acuerdo	*Valoración de resultados*	1. Nada importante	2. Poco importante	3. Importante	4. Muy importante
1. Una alimentación saludable puede reducir mi riesgo de presentar algunas enfermedades (por ejemplo, diabetes, enfermedades del corazón, algunos tipos de cáncer, etc.)	1	2	3	4	5	6	1. ¿Qué tan importante es para mí reducir mi riesgo de enfermedad es?	1	2	3	4
2. Una alimentación saludable puede ayudarme a mejorar mi concentración en la escuela*	1	2	3	4	5	6	2. ¿Qué tan importante es para mí mejorar mi concentraci ón en la escuela?	1	2	3	4
3. Una alimentación saludable puede ayudarme a controlar mi peso	1	2	3	4	5	6	3. ¿Qué tan importante es para mí controlar mi peso?	1	2	3	4
4. Una alimentación saludable puede ayudarme a	1	2	3	4	5	6	4. ¿Qué tan importante es para mí	1	2	3	4

sentirme con más energía durante todo el día*							sentirme con más energía durante todo el día?				

SOCIAL COGNITIVE THEORY - BASED SCALE FOR FACTORS INFLUENCING EATING BEHAVIOR IN ADOLESCENTS

Below are some questions related to your eating habits. Please read the instructions for each section, reflect on the questions, and answer honestly. Remember, THIS IS NOT AN EXAM.

Facilitators<br><br>Circle the number that indicates how much you agree or disagree with each statement.	1. Strongly disagree	2. Disagree	3. Slightly disagree	4. Slightly agree	5. Agree	6. Strongly agree
1. Healthy snacks are available at home.	1	2	3	4	5	6
2. Healthy drinks are available at home (e.g., water, milk, sugar-free beverages).	1	2	3	4	5	6
3. Fruit is always available at home (fresh or dried).	1	2	3	4	5	6
4. Vegetables are always available at home (fresh, cooked, or canned).	1	2	3	4	5	6

Parental Social Support<br><br>Circle the corresponding number for each question.<br><br>*In the last month, how often…	1. Never	2. Rarely	3. Sometimes	4. Frequently	5. Always
1. My parents (or guardians) prepared healthy snacks for me (for example, fruit or unsweetened yogurt).*	1	2	3	4	5
2. My parents (or guardians) prepared healthy meals for me (including breakfasts, lunches, and dinners).	1	2	3	4	5
3. My parents (or guardians) encouraged me to eat fruits and vegetables.	1	2	3	4	5
4. I prepared healthy meals or snacks with my parents (or guardians).	1	2	3	4	5

Peer Social Support<br><br>Circle the corresponding number for each question.<br><br>*In the last month, how often…	1. Never	2. Rarely	3. Sometimes	4. Frequently	5. Always
1. My friends or classmates encouraged me to eat fruits and vegetables.	1	2	3	4	5
2. My friends or classmates encouraged me to drink water, milk, or sugar-free beverages.	1	2	3	4	5
3. My friends or classmates encouraged me to avoid foods high in fat or sugar (fried foods, bread, sweets, sugary drinks).	1	2	3	4	5
4. I ate healthy meals or snacks with my friends or classmates.	1	2	3	4	5

Learning Models<br><br>Circle the number that indicates how much you agree or disagree with each statement.	1. Strongly disagree	2. Disagree	3. Slightly disagree	4. Slightly agree	5. Agree	6. Strongly agree
1. My parents (or guardians) eat healthy foods (for example, fruits or vegetables). *	1	2	3	4	5	6
2. My parents (or guardians) avoid eating foods high in fat or sugar (fried foods, bread, sweets, sugary drinks).	1	2	3	4	5	6
3. My teachers or instructors eat healthy foods (for example, fruits or vegetables).	1	2	3	4	5	6
4. My teachers or instructors avoid eating foods high in fat or sugar (fried foods, bread, sweets, sugary drinks).	1	2	3	4	5	6
5. My friends or classmates eat healthy foods (for example, fruits or vegetables).	1	2	3	4	5	6
6. My friends or classmates avoid eating foods high in fat or sugar (fried foods, bread, sweets, sugary drinks).	1	2	3	4	5	6

Self-Regulation<br><br>Circle the corresponding number for each question.<br><br>*In the last month, how often…	1. Never	2. Rarely	3. Sometimes	4. Frequently	5. Always
1. I chose foods with less fat or healthy fats when available (for example, toast instead of sweet bread, guacamole instead of dressings).	1	2	3	4	5
2. Instead of choosing sugary drinks like soda or bottled juice, I chose plain water or sugar-free drinks (like diet flavored water).	1	2	3	4	5
3. I prepared meals and snacks for myself using healthy, low-sugar ingredients.	1	2	3	4	5
4. I took steps to make fruits and vegetables more enjoyable (for example, trying new recipes and combinations).	1	2	3	4	5

Self-Efficacy<br><br>Circle the number that indicates how much you agree or disagree with each statement.<br><br>When I have to choose a food to eat...	1. Strongly disagree	2. Disagree	3. Slightly disagree	4. Slightly agree	5. Agree	6. Strongly agree
1. I find it easy to choose healthy snacks when I eat between meals (for example, fruit or unsweetened yogurt).	1	2	3	4	5	6
2. I believe I have the knowledge and skills to choose and prepare healthy snacks.	1	2	3	4	5	6
3. I find it easy to choose healthy meals/snacks when I eat out with my friends.	1	2	3	4	5	6
4. I find it easy to avoid foods high in fat or sugar (fried foods, breads, sweets, sugary drinks).	1	2	3	4	5	6
5. I find it easy to eat fruits and vegetables every day.	1	2	3	4	5	6

Circle the number that indicates how much you agree or disagree with each benefit and how important each benefit is to you.											
Outcome Expectations	1. Strongly disagree	2. Disagree	3. Slightly disagree	4. Slightly agree	5. Agree	6. Strongly agree	*Outcome Valuation*	1. Not important	2. Somewhat important	3. Important	4. Very important
1. A healthy diet can reduce my risk of developing some diseases (for example, diabetes, heart disease, some types of cancer, etc.)	1	2	3	4	5	6	1. How important is it for me to reduce my risk of disease?	1	2	3	4
2. A healthy diet can help me improve my concentration at school	1	2	3	4	5	6	2. How important is it for me to improve my concentration at school?	1	2	3	4
3. A healthy diet can help me control my weight	1	2	3	4	5	6	3. How important is it for me to control my weight?	1	2	3	4
4. A healthy diet can help me feel more energetic throughout the day	1	2	3	4	5	6	4. How important is it for me to feel more energetic throughout the day?	1	2	3	4
