# Supplementary material for: Validation of the Revised Version of the Social Cognitive Theory-Based Scale for Factors Influencing Eating Behavior in Adolescents
Source: Healthcare (Basel). 2026 Jul 22;14(14):2229. doi: 10.3390/healthcare14142229 (PMC13411829; doi:10.3390/healthcare14142229)
Supplement: Supplementary file 1 [file healthcare-14-02229-s001.zip › SM 2. 29.06.26.pdf]

SUPPLEMENTARY MATERIAL S2

Characteristics of the items and additive variables in the Social Cognitive Theory - based scale for Factors Influencing Eating Behavior in Adolescents (SCT-FIEBA)

Table S1. Characteristics and measures of skewness and kurtosis of the items in the SCT-FIEBA (*n* = 705).

Item	Range (min-max)	Mean	Standard Deviation	Skewness	Kurtosis
Facilitators 1	1 - 6	4.37	1.53	-0.832	-0.307
Facilitators 2	1 - 6	4.77	1.55	-1.310	.0619
Facilitators 3	1 - 6	4.52	1.62	-0.936	-0.288
Facilitators 4	1 - 6	4.50	1.60	-0.914	-0.302
Parental Social Support 1	1 - 5	3.21	1.07	-0.122	-0.616
Parental Social Support 2	1 - 5	3.64	1.09	-0.531	-0.405
Parental Social Support 3	1 - 5	3.97	1.15	-0.992	0.116
Parental Social Support 4	1 - 5	3.28	1.23	-0.249	-0.895
Self-regulation 1	1 - 5	3.06	1.06	-0.025	-0.542
Self-regulation 2	1 - 5	3.26	1.13	-0.140	-0.738
Self-regulation 3	1 - 5	3.13	1.15	-0.012	-0.830
Self-regulation 4	1 - 5	3.22	1.22	-0.155	-0.952
Self-efficacy 1	1 - 6	4.05	1.39	-0.425	-0.568
Self-efficacy 2	1 - 6	4.23	1.42	-0.604	-0.480
Self-efficacy 3	1 - 6	3.65	1.46	-0.181	-0.906
Self-efficacy 4	1 - 6	3.57	1.49	-0.054	-0.950
Self-efficacy 5	1 - 6	4.39	1.52	-0.677	-0.553
Outcome Expectations 1	1 - 6	4.94	1.55	-1.531	1.160
Outcome Expectations 2	1 – 6	4.77	1.40	-1.164	0.623
Outcome Expectations 3	1 – 6	5.10	1.37	-1.785	2.365
Outcome Expectations 4	1 – 6	5.17	1.31	-1.962	3.269
Outcome Valuation 1	1 – 4	3.43	0.69	-1.090	0.975
Outcome Valuation 2	1 – 4	3.49	0.66	-1.235	1.562
Outcome Valuation 3	1 – 4	3.42	0.76	-1.164	0.730
Outcome Valuation 4	1 – 4	3.47	0.71	-1.288	1.287
Peer Social Support 1	1 – 5	2.26	1.21	0.616	-0.653
Peer Social Support 2	1 – 5	2.52	1.30	0.405	-1.010
Peer Social Support 3	1 – 5	2.30	1.28	0.654	-0.719
Peer Social Support 4	1 – 5	2.91	1.29	0.035	-1.090
Learning Models 1	1 - 6	4.57	1.28	-0.897	0.296
Learning Models 2	1 - 6	4.00	1.39	-0.291	-0.788
Learning Models 3	1 – 6	4.17	1.34	-0.411	-0.549
Learning Models 4	1 – 6	3.93	1.33	-0.316	-0.508
Learning Models 5	1 – 6	3.64	1.42	-0.121	-0.828
Learning Models 6	1 - 6	3.35	1.50	0.067	-0.935

Min-max: minimum and maximum score

Table S2. Characteristics and measures of skewness and kurtosis of the additive variables in the SCT-FIEBA (*n* = 705)

Additive variable	Range (min-max)	Mean	Standard Deviation	Skewness	Kurtosis
Facilitators	4 - 24	18.16	5.52	-1.135	0.438
Parental Social Support	4 - 20	14.09	3.65	-0.498	-0.297
Self-regulation	4 - 20	12.68	3.63	-0.066	-0.530
Self-efficacy	5 - 30	19.90	5.71	-0.384	-0.375
Outcome Expectations	4 - 24	19.98	4.93	-1.769	2.764
Outcome Valuation	4 – 16	13.82	2.27	-1.291	2.014
Peer Social Support	3 – 15	7.08	3.39	-0.498	-0.297
Learning Models	5 - 30	19.09	5.44	-0.087	-0.332

Min-max: minimum and maximum score
